# Analysis of the AMT gene family in chili pepper and the effects of arbuscular mycorrhizal colonization on the expression patterns of *CaAMT2* genes

**DOI:** 10.1186/s12864-023-09226-3

**Published:** 2023-03-29

**Authors:** Lei Fang, Miaomiao Wang, Xiao Chen, Jianrong Zhao, Jianfei Wang, Jianjian Liu

**Affiliations:** 1 College of Resource and Environment, Anhui Science and Technology University, Fengyang, China; 2 MOA Key Laboratory of Plant Nutrition and Fertilization in Lower-Middle Reaches of the Yangtze River, Nanjing Agricultural University, Nanjing, China; 3 Guangdong Key Laboratory for New Technology Research of Vegetables, Vegetable Research Institute, Guangdong Academy of Agricultural Sciences, Guangdong, China

**Keywords:** Ammonium, AMT family, Evolution, Arbuscular mycorrhizal symbiosis, Chili pepper

## Abstract

**Background:**

Ammonium (NH_4_^+^) is a key nitrogen source supporting plant growth and development. Proteins in the ammonium transporter (AMT) family mediate the movement of NH_4_^+^ across the cell membrane. Although several studies have examined AMT genes in various plant species, few studies of the AMT gene family have been conducted in chili pepper.

**Results:**

Here, a total of eight AMT genes were identified in chili pepper, and their exon/intron structures, phylogenetic relationships, and expression patterns in response to arbuscular mycorrhizal (AM) colonization were explored. Synteny analyses among chili pepper, tomato, eggplant, soybean, and Medicago revealed that the *CaAMT2;1*, *CaAMT2.4*, and *CaAMT3;1* have undergone an expansion prior to the divergence of Solanaceae and Leguminosae. The expression of six *AMT2* genes was either up-regulated or down-regulated in response to AM colonization. The expression of *CaAMT2;1/2;2/2;3* and *SlAMT2;1/2;2/2;3* was significantly up-regulated in AM fungi-inoculated roots. A 1,112-bp *CaAMT2;1* promoter fragment and a 1,400-bp *CaAMT2;2* promoter fragment drove the expression of the β-glucuronidase gene in the cortex of AM roots. Evaluation of AM colonization under different NH_4_^+^ concentrations revealed that a sufficient, but not excessive, supply of NH_4_^+^ promotes the growth of chili pepper and the colonization of AM. Furthermore, we demonstrated that *CaAMT2;2* overexpression could mediate NH_4_^+^ uptake in tomato plants.

**Conclusion:**

In sum, our results provide new insights into the evolutionary relationships and functional divergence of chili pepper AMT genes. We also identified putative AMT genes expressed in AM symbiotic roots.

**Supplementary Information:**

The online version contains supplementary material available at 10.1186/s12864-023-09226-3.

## Background

The availability of nitrate (NO_3_^−^) and ammonium (NH_4_^+^) in soil has a substantial effect on the growth of higher plants. NH_4_^+^ transporters (AMTs) in the plasma membrane mediate the uptake of NH_4_^+^ from soil [1, 2]. NH_4_^+^ also accumulates in the cells of leaves through the reduction of NO_3_^−^ [3]. In the root cells, NH_4_^+^ is assimilated in the glutamine synthetase/glutamate synthase cycle, and it is ultimately incorporated into glutamate [4]. In higher plants, NH_4_^+^ can be more efficiently incorporated into glutamate than NO_3_^−^. Consequently, NH_4_^+^ is preferentially assimilated by plants under nitrogen (N)-deficiency conditions [5–7]. However, excessive NH_4_^+^ concentrations can have toxic effects on various plants [8]. There are two transport systems for ammonium uptake, the high-affinity transport system, which is the main AMT under low external NH_4_^+^ concentrations (< 0.5 mM), and the low-affinity transport system, which mediates NH_4_^+^ uptake under high external NH_4_^+^ concentrations (> 0.5 mM) [9, 10].

Arbuscular mycorrhizal (AM) symbioses are some of the oldest and most important symbiotic systems in natural soil ecosystems, as more than 80% of land plants are engaged in symbiotic associations with AM fungi from the subdivision Glomeromycotina [11–13]. Symbiotic associations with AM fungi not only increase the uptake of nutrients, including phosphorus (P), N, and potassium (K), but also enhance the resistance of plants to abiotic and biotic stress, such as drought, salinity, and pathogen infection [14–18]. The availability of mineral nutrients in host plants and soil also affects interactions with AM fungi. For example, high phosphate (Pi) concentrations impede AM colonization [19, 20]. In tomato, low concentrations of K^+^ have a negative effect on the AM colonization rate and the incidence of arbuscules [16]. AM colonization is favored when the supply of N is sufficient, not excessive. In AM fungi-inoculated roots of *Andropogon gerardii*, AM colonization and the incidence of arbuscules were highest under 1.5 mM NO_3_^−^ [21]. Low NO_3_^−^ application results in a reduction of percent root length colonization in rice and sorghum plants [17].

The functions of AMTs in response to AM colonization have been characterized in several plant species. The sorghum AMTs *SbAMT3;1* and *SbAMT4* are expressed in AM-colonized root cortical cells and are localized to the periarbuscular membrane [22]. In *Latus japonicus*, the expression of *LjAMT2;2* (high-affinity AMT gene) is highly up-regulated in root cells containing arbuscules [18, 23]. The expression of five AMT genes in the soybean genome are up-regulated by AM symbiosis, including *GmAMT1;4*, *GmAMT3;1*, *GmAMT4.;1*, *GmAMT4;3*, and *GmAMT4;4*. The *LjAMT2;2* ortholog *GmAMT4.;1* is specifically expressed in arbuscular cells but not in membranes surrounding arbuscular trunks [24]. In *Medicago truncatula*, the expression of three AMT2 proteins is induced by AM colonization, and MtAMT2;3 is required for the suppression of premature arbuscular degeneration in *mtpt4* mutants [25]. The role of AM fungi in NO_3_^−^ and NH_4_^+^ uptake has also been clarified. NH_4_^+^ is the preferred N source for AM fungi because additional energy is required for the reduction of NO_3_^−^ to NH_4_^+^ by nitrate and nitrite reductase [5, 26]. The three ammonium transporter genes *GinAMT1*, *GinAMT2*, and *GinAMT3* have been identified in *Rhizophagus irregularis*. GintAMT1 and GintAMT2 show high-affinity NH_4_^+^ transport activity in a yeast AMT triple-deletion mutant and are expressed in the extraradical mycelium (ERM) [27–29]. The genes encoding these two AMTs have been shown to have differential expression patterns in response to changes in the NH_4_^+^ supply. For example, the expression of *GintAMT1* is down-regulated after the supply of NH_4_^+^ is restored, but the expression of *GintAMT2* is up-regulated following restoration of the supply of NO_3_^−^ and NH_4_^+^ under low-N conditions. GintAMT3 shows low-affinity transport activity, and it is constitutively expressed in ERM and intraradical mycelium [28]. AMT genes have been studied in various plant species, including Arabidopsis, rice, wheat, poplar, tomato, and *Pinus pinaster* [3, 30–35]. Three AMT genes have been isolated from cDNA libraries of the roots and leaves in tomato, and these three AMTs could functionally complement a yeast mutant with impaired NH_4_^+^ uptake. The high-affinity AMTs LeAMT1;1 and LeAMT1;2 might be involved in root hair-mediated NH_4_^+^ uptake from soil [6].

Chili pepper is an economically important vegetable crop that is rich in vitamins, carotenoids, and capsaicinoids; it is used extensively as a spice, food, and pharmaceutical product [36, 37]. In 2020, chili pepper was cultivated on more than 2 million hectares, and approximately 36.2 million metric tons of chili pepper were produced in worldwide (FAO, 2020). NH_4_^+^ application significantly increases chili pepper yields and enhances the accumulation of capsaicin and dihyfrocapaicin in fruits [38]. However, the role of NH_4_^+^ in pepper has received less attention, and no study has examined the relationship between NH_4_^+^ and AM symbiosis. The release of the chili pepper genome sequence has now made it possible to elucidate the molecular mechanisms underlying NH_4_^+^ acquisition and AM symbiosis in chili pepper [39].

Here, we identified eight AMT genes (*CaAMTs*) from chili pepper using comparative genomic tools and experimentally verified the identity of these genes. We analyzed the phylogenetic relationships of these genes, their exon/intron structures, and their expression patterns in response to AM colonization. We also studied the efficiency of AM symbiosis under a low and high supply of NH_4_^+^. The results of our study provide new insights into the mechanisms driving the evolution of AMT genes in chili pepper. Our results suggest that mycorrhiza-inducible AMT genes might play an important role in symbiotic N uptake in chili pepper and other higher plants.

## Results

### Identification of AMT genes in the chili pepper genome

To identify candidate AMT genes in the chili pepper genome, the amino acid (AA) sequences of homologous AMT genes in *Arabidopsis* were used to conduct a BLAST search against the chili pepper protein database. A total of eight AMT proteins with an Ammonium_transp domain (PF09009) were identified using the SMART and Pfam databases. The AMT genes in chili pepper were named according to homologous AMT genes in tomato and *Arabidopsis*. Basic information on these genes, including gene IDs, as well as the lengths, MW, and pI of their corresponding proteins, are listed in Table S1. The length of CaAMT proteins ranged from 461 (CaAMT1;1) to 513 (CaAMT1;2) AAs, and the predicted MW ranged from 49.9 to 55 kDa. The pI of these CaAMTs ranged from 5.44 to 8.53.

The two conserved domains DFAGSGVVHnVGGnAGnnG(n)3EGPR and D(n)2GGYVH(n)3GnAGnFAAWVGPR, which have been suggested to be present in AMT genes in angiosperms and gymnosperms [3], were identified in AMT1 and AMT2 proteins, respectively, using the alignment tools in MEGA 7.0 software (Fig. S1). The transmembrane (TM) domain predicted by the TMME2.0 web server suggested that all CaAMT proteins possess the conserved structures of 11 TM domains (Fig. S2). These findings indicated that all the genes identified in the chili pepper genome were AMT genes.

### Chromosomal distribution of AMT genes in the chili pepper genome and AMT gene expansions in solanaceous plants

**Fig. 1:**
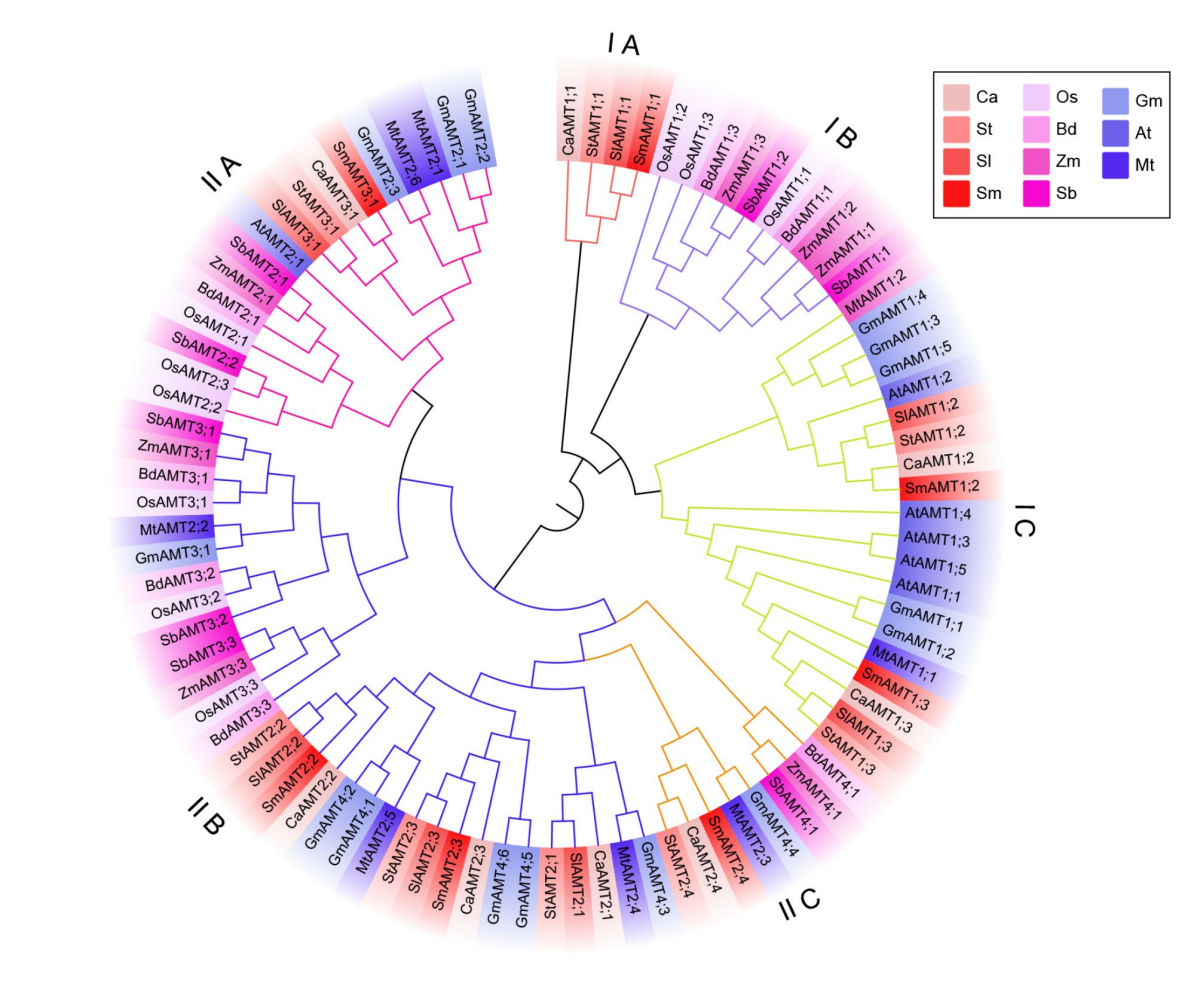
The phylogenetic analysis of eight chili pepper AMT genes and other plant AMT homologs. A total of 91 AMT proteins from *Arabidopsis* (6 *AtAMT*), rice (10 *OsAMT*), *Sorghum bicolor* (8 *SbAMT*), maize (7 *ZmAMT*), *Brachypodium distachyon* (7 BdAMT), tomato (7 *SlAMT*), chili pepper (8 *CaAMT*), potato (8 *StAMT*), eggplant (7 *SmAMT*), soybean (15 *GmAMT*), *Medicago* (8 *MtAMT*) were aligned using ClustalW, and unrooted phylogenetic tree was constructed using the Neighbor-Joining method with 1000 bootstrap repetitions within the MEGA 7.0 software. The AM-induced AMT genes are highlighted with red color in the phylogenetic tree

A chromosome map was constructed to compare the coding sequences of CaAMT genes against the chili pepper genome database. *CaAMT* genes were identified on five chili pepper chromosomes (Fig. S3); chromosomes 10 and 12 had only one AMT gene (*CaAMT3;1* and *CaAMT1;2*, respectively), chromosomes 3, 4, and 8 contained two AMT genes (*CaAMT1;3/2.3*, *CaAMT1;1/2.4*, and *CaAMT2;1/2.2*, respectively). A phylogenetic tree was constructed using the N-J method in MEGA 7 software to clarify the evolutionary relationships among AMT genes. A total of 91 AMT genes from 10 species in four families, Brassicaceae (6 from *Arabidopsis*), Gramineae (10 from rice, 7 from maize, and 8 from *Brachypodium distachyon*), Leguminosae (15 from soybean and 8 from *M. truncatula*), and Solanaceae (7 from tomato, 7 from eggplant, 8 from potato, and 8 from chili pepper), were used in the phylogenetic analysis. The 85 AMT genes were classified into two major groups (Fig. 1). Group I comprised three subgroups, one Solanaceae-specific subgroup, one Gramineae-specific subgroup, and one dicot-specific subgroup, which were referred to as IA, IB, and IC, respectively. In chili pepper, *CaAMT1;1* was in the IA subgroup, and *CaAMT1;2* and *CaAMT1;3* were in the IC subgroup. The five *CaAMT* genes within Group II comprised three subgroups. The three chili pepper paralogs, *CaAMT2;1*, *CaAMT2;2*, and *CaAMT2;3*, which share several AAs, were in subgroup IIB. *CaAMT3;1* was in subgroup IIA. Subgroup IIC only contained AMT genes from the families Leguminosae and Solanaceae, including *CaAMT2.4*.

**Fig. 2:**
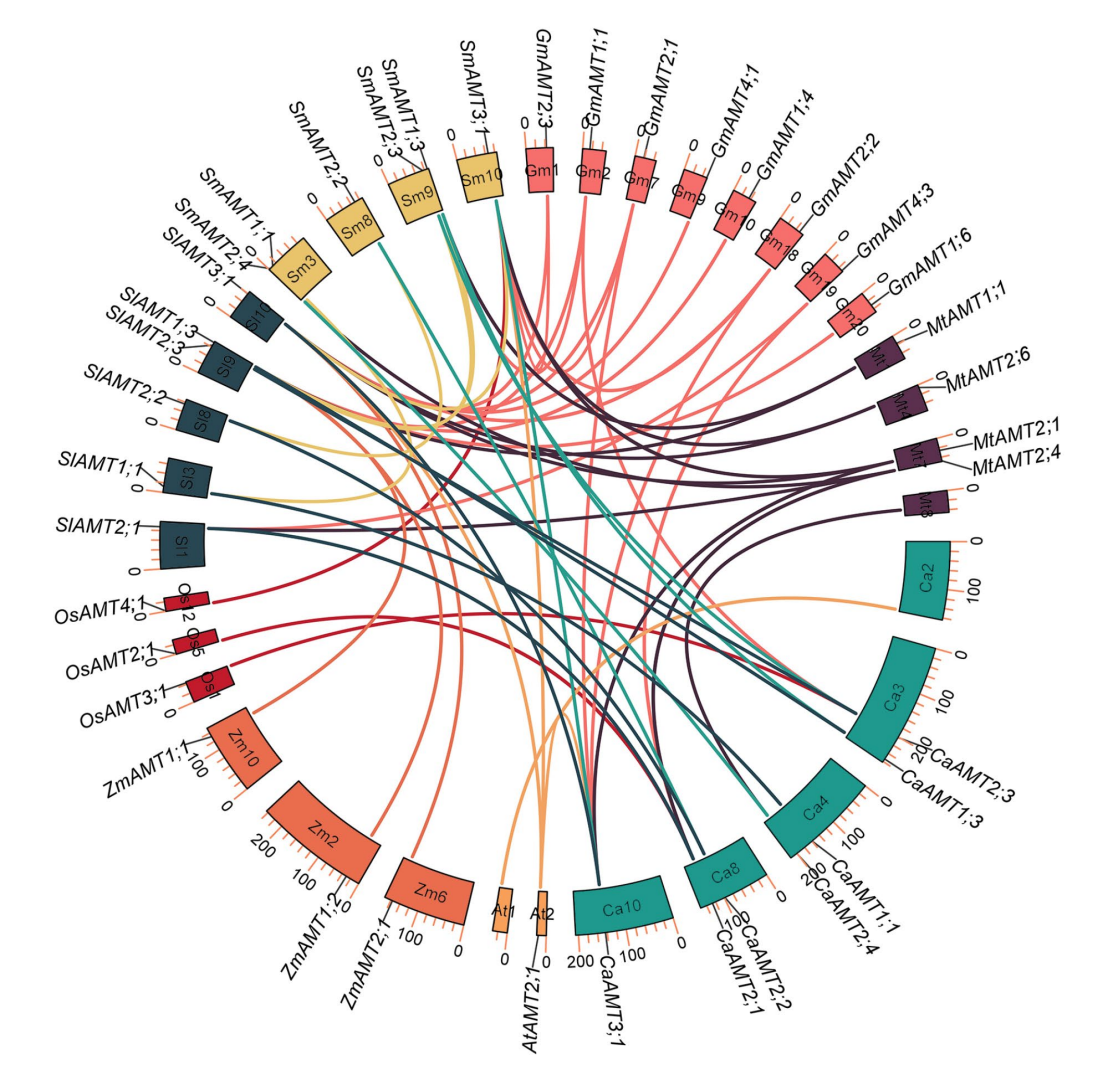
Synteny analyses between the AMT genes of chili pepper and three plant families (Solanaceae, Leguminosae, and Gramineae). The synteny relationships among the AMT genes of chili pepper and three plant families were constructed by Multiple Synteny Plot program within TBtools v.1.0983 software. Gray lines indicated collinear blocks within the chili pepper and other plant species genome. The synteny AMT gene pairs between chili pepper and other six plant species were highlighted with blue lines

The syntenic relationships of CaAMTs with orthologs in three plant families, including Solanaceae (tomato and eggplant), Leguminosae (soybean and *M. truncatula*), and Gramineae (rice and *Sorghum bicolor*) were studied (Fig. 2). In Solanaceae, a total of 12 pairs of orthologous genes, including 6 *SmAMTs* and 6 *SlAMTs*, were identified in eggplant and tomato, respectively. All AMT orthologs in tomato and eggplant were in the same physical positions on homologous chromosomes, with the exception of *SlAMT1;2* and *SmAMT1.2*, which were detected on chromosome 4 and chromosome 11, respectively; however, high conservation of the chromosomal location of AMT genes was not observed in the chili pepper genome. This finding suggests that these orthologous genes might have originated from tandem duplications during the evolution of tomato and eggplant AMT genes. In Gramineae, five and three pairs of orthologous genes were detected between chili pepper and soybean and between chili pepper and *Medicago*, respectively. No orthologous genes were identified between chili pepper and *S. bicolor* and between chili pepper and rice, which suggests that the functional divergence of *CaAMTs* and *OsAMTs/SbAMTs* might have occurred after the divergence between chili pepper and rice and between chili pepper and *S. bicolor*. Orthologous genes of *CaAMT2;1*, *CaAMT2.4*, and *CaAMT3;1* were detected in the tomato, eggplant, soybean, and *Medicago* genomes, which suggests that the expansion of these three genes might have occurred prior to the divergence of Solanaceae and Leguminosae.

### Effects of different levels of NH_4_^+^ on plant N uptake and AM colonization

**Fig. 3:**
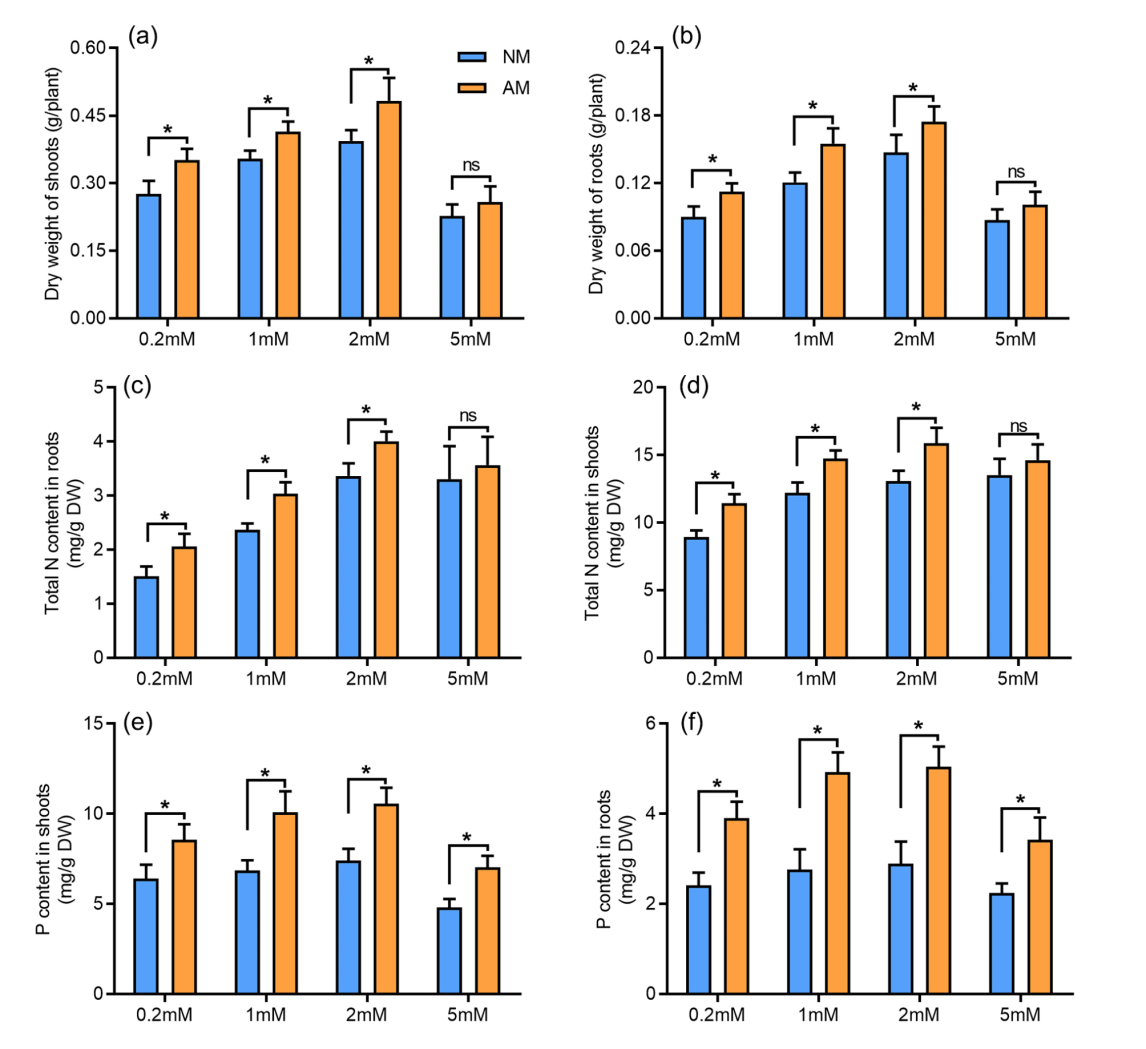
Effects of AM fungal colonization on chili pepper biomass (dry weight, DW), N and P acquisition under different NH_4_^+^ supply conditions. a and b, Shoot (a) and (b) root biomass. c-f, N and P contents in shoots (d, e) and roots (c, f). The chili pepper plants were inoculated with *Rhizophagus irregularis*, and grown under 0.2 mM, 1 mM, 2 mM, 5 mM NH_4_^+^ supply conditions for 5 weeks. AM, mycorrhizal plants; NM, nonmycorrhizal plants. Values are means ± SE of six biological replicates from two pots (n = 6). The asterisks indicate significant differences. *P < 0.05, ns, no significant difference

To characterize the sensitivity of AM fungi-inoculated chili pepper plants to NH_4_^+^ levels, chili pepper plants were grown in a pot culture system with or without *R. irregularis* inoculation and supplemented with either 0.2, 1, 2, or 5 mM NH_4_^+^ weekly. The chili pepper shoots and roots were harvested 5 weeks after *R. irregularis* inoculation for measurements of biomass, N accumulation, and AM colonization rates. The growth of the roots and shoots and N accumulation were significantly increased in AM fungi-inoculated plants treated with 0.2, 1, and 2 mM NH_4_^+^ compared with AM fungi-uninoculated plants; no significant difference in root biomass was observed between AM fungi-inoculated and AM fungi-uninoculated plants in the 5 mM NH_4_^+^ treatment (Fig. 3). The growth of chili pepper plants was reduced in the 5 mM NH_4_^+^ treatment compared with the other NH_4_^+^ levels, indicating that plants experienced high NH_4_^+^ stress (Fig. 3a, b). The N content of AM-uninoculated plants increased as the NH_4_^+^ supply increased in the 0.2, 1, and 2 mM NH_4_^+^ treatments. No significant differences in the N content of the shoots and roots were observed between AM-inoculated and AM-uninoculated plants in the 5 mM NH_4_^+^ treatment. The total N content of both the shoots and roots was significantly higher in AM-inoculated plants than in AM-uninoculated plants in the 0.2, 1, and 2 mM NH_4_^+^ treatments.

**Fig. 4:**
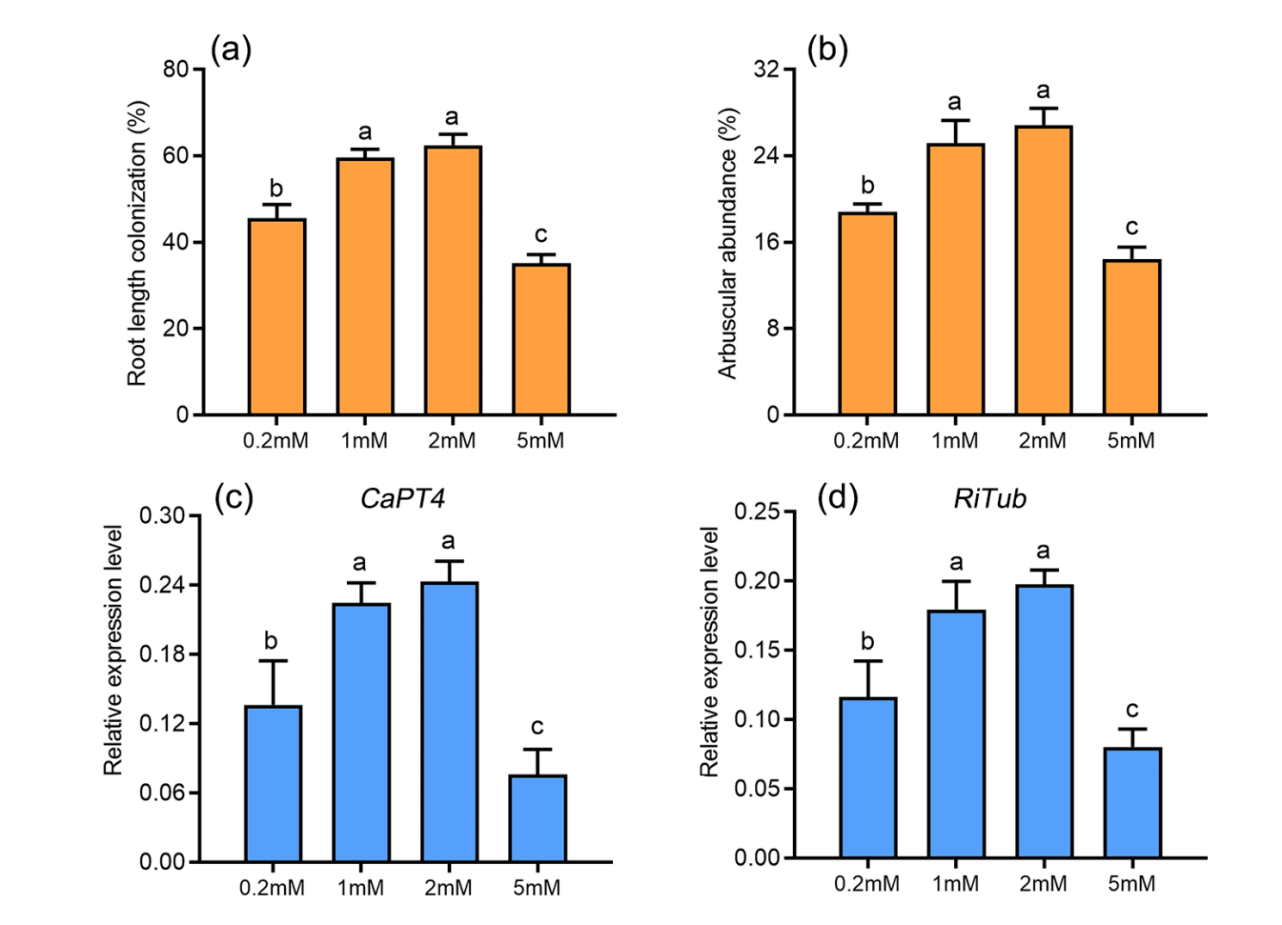
Effects of different concentrations of NH_4_^+^ supply on the mycorrhizal colonization and the expression of AM-marker genes. a and b, mycorrhizal colonization level (a) and arbuscule abundance (b) in mycorrhizal roots of chili pepper plants were determined. c and d, the transcript levels of *CaPT4* (c) and *RiTub* (d) in chili pepper inoculated or non-inoculated with *Rhizophagus irregularis. CaPT4* and *RiTub* from chili pepper and *R. irregularis*, respectively, were used for evaluating AM colonization. Values are means ± SE of six biological replicates from two pots (n = 6). Different letters indicate significant differences, *P* < 0.05

The concentration of P was higher in the shoots and roots in AM-inoculated chili pepper plants than in AM-uninoculated chili pepper plants, regardless of the amount of N supplied (Fig. 3e, f). AM colonization efficiency under different NH_4_^+^ levels was evaluated by measuring percent root length colonization and arbuscular abundance Root colonization and arbuscules abundance were reduced in AM-inoculated plants under low and high NH_4_^+^ concentrations (Fig. 4a, b). The reduced root colonization under low and high NH_4_^+^ concentrations was also associated with down-regulated expression of the two AM-specific marker genes *CaPT4* and *RiTub* (Fig. 4c, d).

### Contribution of the mycorrhizal pathway to NH_4_^+^ uptake in chili pepper plants

**Fig. 5:**
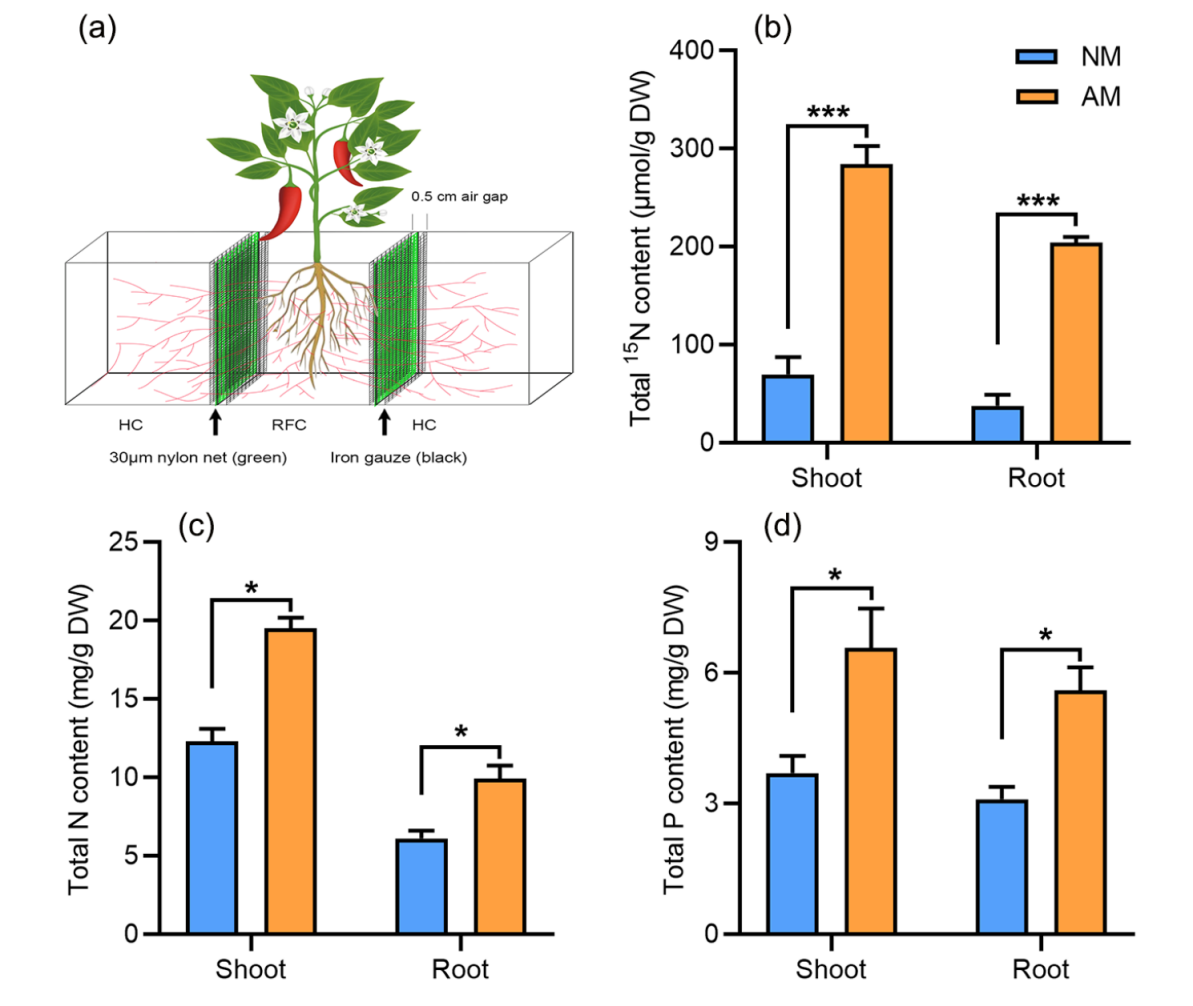
Effects of the mycorrhizal pathway on chili pepper NH_4_^+^ uptake. (a) A diagram representing the compartmented growth systems; (b) ^15^ N signal in chili pepper shoots and roots grown with or without AM fungi. (c, d) Total P (c) and N (d) content of chili pepper plants. RFC: plant chamber, HC: hyphal chamber. Error bars indicate SE (n = 6). Student’s t-test (*P < 0.05, ***P < 0.001) was used to analysis statistical significance; ns, not significant

The mycorrhizal pathway has been shown to contributed to the uptake of essential nutrients (e.g. Pi, N and K) by plants [12]. In our study, a compartmentalized culture system was used to determine whether the mycorrhizal uptake pathway was essential for NH_4_^+^ acquisition in chili pepper plants (Fig. 5a). Chili pepper plants inoculated or non-inoculated with AM fungi were transplanted to the middle compartment (RFC), and ammonium sulfate-^15^ N was added to the hyphal compartments (HCs). After 5-weeks since inoculation, we determined the ^15^ N abundance, and the N/P content in the shoots and roots of chili pepper plants. As shown in Fig. 5b, ^15^ N accumulation in both of shoots and roots of mycorrhizal chili pepper plants was higher than that in the non-inoculated plants. The same trend was observed for the total N content of mycorrhizal plants (Fig. 5c). In addition, mycorrhizal plants showed a significantly increased P content in both shoots and roots, compared with those in the non-inoculated plants (Fig. 5d). Therefore, our finding demonstrated that the mycorrhizal pathway contributes to NH_4_^+^ acquisition in chili pepper.

### CaAMT2 expression responds to colonization by AM fungi

**Fig. 6:**
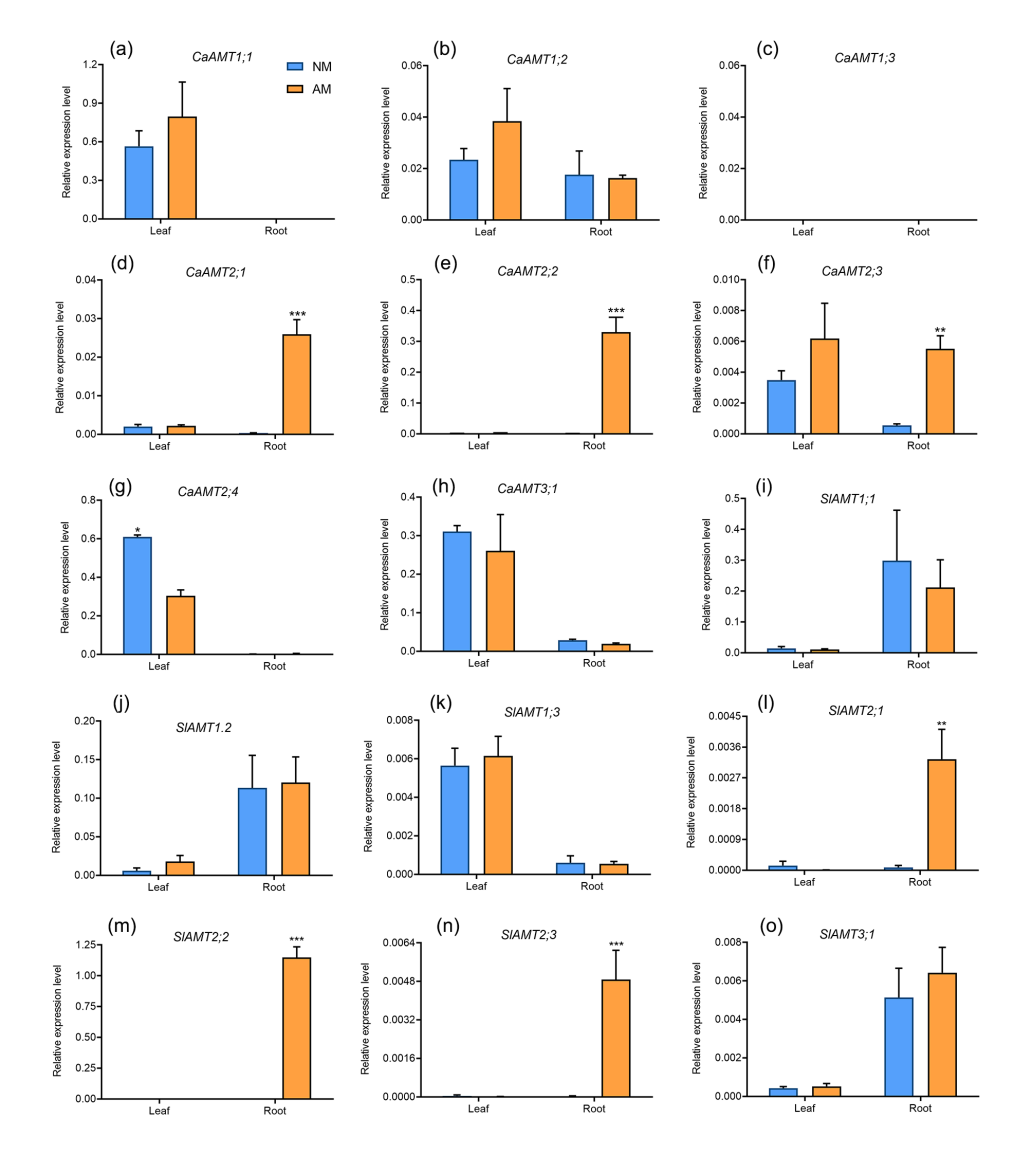
Transcriptional regulation of the chili pepper and tomato AMT genes in response to AM fungal colonization. AM, mycorrhizal plants; NM, nonmycorrhizal plants. Values are means ± SE of three biological replicates (n = 3). The asterisks indicate significant differences. *P < 0.05, ***P < 0.001

**Fig. 7:**
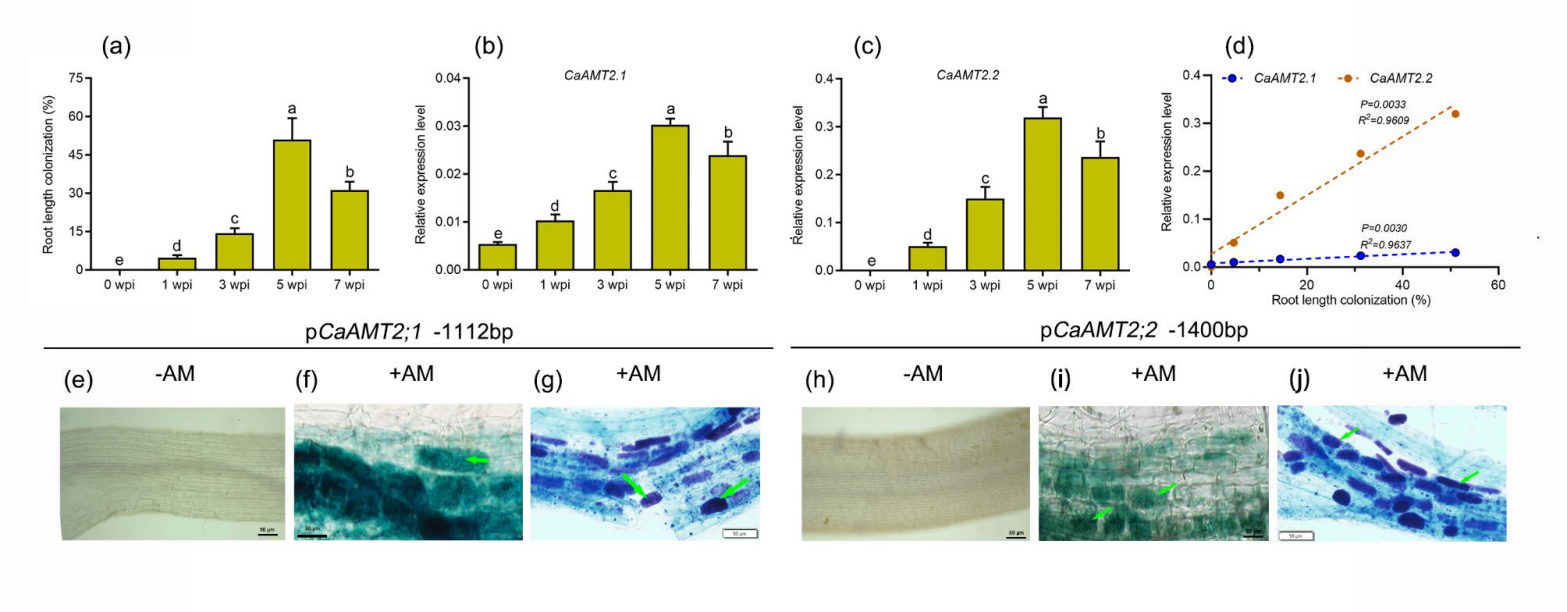
Expression analysis of *CaAMT2;1* and *CaAMT2;2* in response to AM symbiosis. (a) Quantification of AM colonization at different sampling time points. Wpi, Weeks post-inoculation. (b-c) Time-course expression patterns analysis for *CaAMT2;1* and *CaAMT2;2*. (d) The correlation analysis of AM colonization and CaAMT2;1/CaAMT2;2 expression level. (e, h) No GUS staining was detected in the roots expressing p*CaAMT2;1*::GUS and p*CaAMT2;2*::GUS in the absence of inoculation. (f, i) Blue GUS staining directed by CaAMT2;1 and CaAMT2;2 promoter in tomato mycorrhizal roots. (g, j) Co-localization of GUS activity (indicated by the purple color, from the overlay of the Magenta-GUS and Trypan Blue stains). Blue arrows indicate arbuscules

AMT genes have been shown to be regulated by AM colonization in several studies. The expression levels of seven *CaAMT* genes in the leaves and roots of AM fungi-inoculated and AM fungi-uninoculated plants were examined using RT-PCR analysis to clarify the functions of *CaAMT* genes in response to AM colonization. The expression of Group I genes, including *CaAMT1;1* and *CaAMT1;2*, did not significantly change in response to AM colonization (Fig. 6a, b). The expression of Group IIA genes, including *CaAMT2;1* and *CaAMT2;2*, was low or absent in the leaves and roots of AM fungi-uninoculated plants but was significantly increased in AM fungi-inoculated roots (Fig. 6d, e). The expression level of *CaAMT2;3* in roots was also upregulated by AM fungal colonization (Fig. 6f). However, the expression of *CaAMT2.4* in leaves was down-regulated by AM fungal colonization (Fig. 6g). No significant changes in the expression of *CaAMT3;1* between AM fungi-inoculated and AM fungi-uninoculated plants were observed (Fig. 6h). Additional expression time-course experiments were performed to characterize the effects of AM symbiosis on the expression of *CaAMT2s*. The levels of *CaAMT2;1* and *CaAMT2;2* expression were highly correlated with AM fungal colonization (Fig. 7d).

The expression patterns of *AMT2* orthologs in tomato were also analyzed to determine whether the expression of *AMT2* orthologs in other solanaceous species is induced by AM colonization. The expression of *SlAMT2* genes was highly up-regulated in AM fungi-inoculated roots (Fig. 6l-n). These findings suggest that *AMT2* genes have played an important role in regulating the colonization of AM fungi in several solanaceous plants.

### **The promoters of *CaAMT2;1* and *CaAMT2;2* can drive the expression of AM-specific genes in the roots of AM fungi**

Several AM-specific transporters, such as *MtPT4*, *OsPT11*, *OsNFP4.5*, and *SlHAK10*, required for the acquisition of nutrients via AM pathways, are specifically expressed in cells containing AM fungal structures. A 1,112-bp promoter fragment of the *CaAMT2;1* gene and a 1,400-bp promoter fragment of the *CaAMT2;2* gene were fused to the GUS reporter gene and then introduced into chili pepper plants to characterize the expression of *CaAMT2s* in AM fungi-inoculated roots. Transgenic chili pepper roots were harvested five weeks following inoculation with *R. irregularis*. GUS activity in chili pepper roots expressing *CaAMT2;1* and *CaAMT2;2* was not detected in AM fungi-uninoculated plants; by contrast, co-localization of GUS expression and AM structures via overlay of Magenta-GUS with trypan blue staining revealed that GUS activity driven by the *CaAMT2;1* and *CaAMT2;2* promoters was confined to cortical cells containing arbuscules **(**Fig. 7e-h).

### **Ectopic expression of *CaAMT2;2* enhances NH**_**4**_^**+**^** uptake in tomato**

**Fig. 8:**
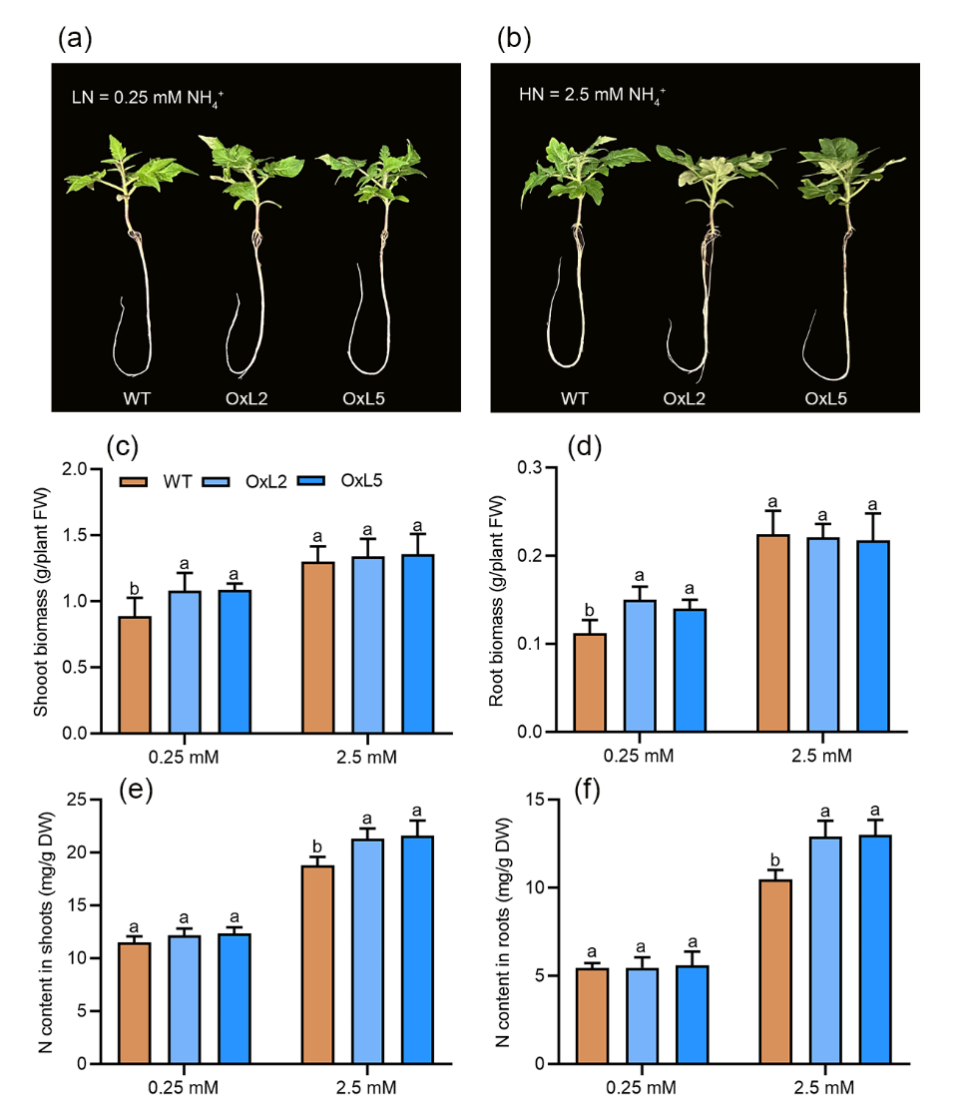
CaAMT2;2 mediates the N uptake in transgenic tomato plants. (a, b) Growth performance of the wild-type (WT) plants and *CaAMT2;2*-overexpressing transgenic lines under deficient (0.25 mM NH_4_^+^) and NH_4_^+^-sufficient (2.5 mM NH_4_^+^) conditions. Two-week-old seedings were grown in the full nutrient solution for 2 weeks, and then transferred to different NH_4_^+^ solutions for 2 weeks. (c, d) Shoot and root biomass (fresh weight, FW) of tomato plants. (e, f) Total N content in the shoots and the roots. Values are means ± SE of six biological replicates (n = 6). Different letters indicate significant differences, *P* < 0.05

To investigate the role of CaAMTs in NH_4_^+^ uptake, we ectopically overexpressed *CaAMT2;2* in tomato. Two independent lines (OxL2 and OxL5) that showed high *CaAMT2;2* expression were selected for analysis of NH_4_^+^ absorption in a hydroponic system containing either 0.25 mM NH_4_^+^ or 2.5 mM NH_4_^+^ conditions (Fig. S4a). After 12 d of cultivation, shoot and root biomass was significantly higher in Ox lines than in wildtype (WT) plants under 0.25 mM NH_4_^+^ conditions. Shoot and root N accumulation was also higher in Ox lines than in WT plants under 2.5 mM NH_4_^+^ conditions (Fig. 8). Although the N content did not differ significantly between Ox lines and WT plants grown under 0.25 mM NH_4_^+^ conditions, N accumulation was significantly higher in Ox lines than in WT plants (Fig. S4b, c). The finding suggests that CaAMT2;2 could enhance NH_4_^+^ uptake in tomato.

## Discussion

NH_4_^+^ is a key N source for the growth and development of plants. Several *AMT* genes have been identified in many plant species, including *Arabidopsis thaliana*, *Oryza sativa*, maize, soybean, and *Medicago* [25, 30, 40]. Genome-wide studies of *AMT* genes in chili pepper plants are lacking. In this study, seven *AMT* genes were identified, and these *AMT* genes were divided into two groups: three of these *AMT* genes were in Group I, and the other four *AMT* genes were in Group II. The total number and distribution of *AMT* genes were highly conserved in chili pepper, potato, tomato, and eggplant, suggesting that the functions of *AMT* genes required for NH_4_^+^ acquisition and metabolism are conserved among solanaceous plants. Two highly conserved domains were detected in different species, which is consistent with findings in other plant species, such as *P. pinaster*, *Picea glauca*, and *Populus trichocarpa* [3]. The conservation of gene function might be associated with the conservation of gene structure. In *Populus* and *Lotus japonicus*, most *AMT1* genes have only one exon, with the exception of *LjAMT1;1* and *PtAMT1;7*, which have one intron [34, 41]. The structure of chili pepper AMT genes is also highly conserved; for example, among *CaAMT1* genes, *CaAMT1;1* is the only gene with an intron, as *CaAMT1;2* and *CaAMT1;3* lack introns. Von Wittgenstein et al. (2010, 2012) showed that *AMT1* genes in plants were inherited vertically from a common ancient ancestor, and the divergence of these genes predated the split between bryophytes and embryophytes but occurred after the separation of land plants and green algae [41, 42].

Although the numbers of introns in *AMT2* genes ranged from 0 to 4, some exons (285 bp) are shared among *AMT2* homologs in Solanaceae, Gramineae, and Leguminosae. Synteny analysis among different genomes is important for clarifying the rapid evolution of gene families. Collinearity analysis revealed 36 homologous gene pairs among *AMT* genes from chili pepper, *S. bicolor*, rice, tomato, eggplant, soybean, and *Medicago* at the genome-wide scale (Fig. 2), and these genes were classified into four groups with high sequence similarity. The *CaAMT3;1* gene in group IV was the most highly conserved. Synteny analysis of *CaAMT* genes between chili pepper and each of the six other plant species revealed that the number of collinear gene pairs of *AMT* genes was highest in Solanaceae, and the lowest number of collinear gene pairs was observed in Gramineae. These findings indicate that duplication of *CaAMT* genes might have occurred following the divergence between Solanaceae and Gramineae.

AM symbiosis plays a key role in mediating the uptake of mineral nutrients in many land plants. Although the mechanism of N uptake and transport via AM fungi has received less attention compared with that of Pi, the key role of AM symbiosis in N accumulation in plants has been demonstrated in several plant species [43–45]. In the soil, the ERM of AM fungi can absorb both organic and inorganic N, such as NH_4_^+^, NO_3_^−^, and AAs [15, 46, 47]. AM fungi-inoculated maize plants have been shown to absorb 10 times as much ^15^ N from NH_4_^+^ than NO_3_^−^ in a two-compartment growth system [48]. Wang et al. (2020) showed that AM fungi-inoculated rice receives more than 40% of its N via AM pathways [17]. We found that AM symbiosis could promote the growth of chili pepper under the 0.2 mM, 1 mM, and 2 mM NH_4_^+^ treatments, but not for the 5 mM NH_4_^+^ treatment. The toxicity associated with an excess NH_4_^+^ supply is the main factor underlying the inhibition of plant growth and the AM colonization rate (Fig. 3a, b). N accumulation was higher in the shoots and roots of AM fungi-inoculated plants than in AM fungi-uninoculated plants in all treatments, with the exception of the 5 mM NH_4_^+^ treatment. Recently, Hui et al. (2022) identified a mycorrhizal-specific AMT gene, ZmAMT3;1, that mediates N transfer from mycorrhizal fungi to maize plants under pot-culture and field-grown conditions [49]. In this study, the mycorrhizal pathway was also shown to be essential for N acquisition in chili pepper in a compartmentalized culture system (Fig. 5).

The high availability of mineral nutrients in rhizosphere soil, especially the high Pi concentrations, has a strong negative effect on AM colonization [20, 25]. The efficiency of AM symbiosis of tomato and *Lycium barbarum* is enhanced by K^+^ application; however, in *M. truncatula*, the availability of K has no effect on AM colonization [16, 50, 51]. Our findings indicate that a sufficient supply of N is essential for maintaining the functions of AM symbiosis. The AM colonization rate of *Petunia hybrida* is higher under 5 mM NO_3_^−^ treatment than under 1.9 mM NO_3_^−^ treatment, but the AM colonization rate is significantly reduced when the NO_3_^−^ supply increases (19 mM NO_3_^−^) [21]. In rice and sorghum, a significant decrease in the AM colonization rate and the expression of AM-specific marker genes under low NO_3_^−^ treatment indicates that N plays a key role in AM symbiosis-induced host metabolic changes [13, 17]. Recently, Wang et al. found that the mycorrhizal colonization rate was higher under 1.0 mM NH_4_^+^ conditions than under 0.05 mM and 0.5 mM NH_4_^+^ conditions in *Lotus japonicus* [18]. In this study, we observed a significant increase in AM colonization when the NH_4_^+^ supply was sufficient, and AM colonization was reduced in the high ammonium (5 mM NH_4_^+^) treatment (Fig. 4). In *Lactuca sativa* and cowpea plants, excessive NH_4_^+^ concentrations have been shown to inhibit primary root growth and have direct deleterious effects on the ERM [52, 53].

AM colonization has been shown to induce the expression of *AMT* genes, such as *MtAMT2;3/2;4/2;5*, *SbAMT3;1/4*, and *OsAMT3;1*, in *Medicago*, *S. bicolor*, and rice, respectively [22, 25, 54]. In our study, expression analysis of eight CaAMT genes revealed that the expression of three *AMT2* genes, *CaAMT2;1*, *CaAMT2;2*, and *CaAMT2;3*, was strongly induced by AM colonization. The expression of three tomato AMT paralogs, *SlAMT2;1*, *SlAMT2;2*, and *SlAMT2;3*, was also induced by AM colonization (Fig. 6). These results indicated that AM symbiosis plays an important role in regulating AMT expression. Chili pepper and tomato plants contain a relatively high proportion of AM-activated *AMT2* genes, which suggests that some of the *AMT2* gene family members within the family Solanaceae are functionally redundant. Furthermore, nearly all AM fungi-induced AMT genes were in Group II, with the exception of *GmAMT1.4*, which suggests that the duplication events underlying the origin of AM fungi-induced *AMT2* genes in Solanaceae, Leguminosae, and Gramineae predated the monocot–dicot split. The AA sequences of *AMT* genes from Group II differed from those in Group I, which reflects their distinct evolutionary origins (Table S2). McDonald et al. (2012) found that *AMT2* genes in land plants are sister to a group of AMT2 genes in lichenized Ascomycota and argued that the *AMT2* genes of land plants might have arisen through ancient independent horizontal gene transfer events from Archaea and gamma proteobacteria [41]. This indicates that *AMT2* genes have played an important role in AM symbiosis during the evolution of land plants.

Structures of AM fungi that develop from highly differentiated fungal hyphae are the site of nutrient and signal molecule exchange between plants and fungi. Multiple genes have been shown to be involved in the transport of nutrients, especially in P nutrient, and these genes are specifically expressed in root cells hosting arbuscules and play a key role in regulating AM symbiosis [55–57]. In our study, strong GUS activity driven by the *CaAMT2;1* and *CaAMT2;2* promoter regions (*pCaAMT2;1*_*− 1112*_ and *pCaAMT2;2*_*− 1400*_, respectively) was detected in arbuscule-containing cells (Fig. 7f, h). Multiple genes involved in nutrient transport, including the transport Pi, NH_4_^+^/NO_3_^−^, and K have been characterized in previous studies, and these studies have shown that these genes are specifically expressed in arbuscules-containing root cells and play a crucial role in nutrient absorption or in modulating AM symbiosis [16, 55, 58, 59]. Recently, overexpression of a mycorrhizal-induced ammonium transporter gene, *LjAMT2;2* has been shown to significantly increase plant growth and N uptake under both NH_4_^+^-deficient and NH_4_^+^-sufficient conditions [18].

## Conclusion

This is the most comprehensive study to date of the *AMT* gene family in chili pepper. We characterized the phylogenetic relationships among *AMT* genes, as well as their exon/intron structures, conserved domains, and AM-specific transcriptional profiles. The expression of *CaAMT2;1* and *CaAMT2;2* was highly up-regulated in response to AM colonization, and GUS activity analysis revealed that these two genes were specifically expressed in AM fungi-colonized root cells. We also studied the effect of NH_4_^+^ levels on the efficiency of AM symbiosis in chili pepper plants, and demonstrated that CaAMT2;2 could mediate NH_4_^+^ uptake in tomato plants. Our findings provide new insights into the evolution of *AMT2* genes under AM symbiosis. The two key candidates *AMT2* genes identified in our study could potentially represent transporter genes activated by AM symbiosis. Additional studies of the functional complementation of yeast AMT mutants and the physiological phenotypes of mutants containing these *AMT2* genes in chili pepper are needed.

## Methods

### Plant material and growth conditions

The seeds of chili pepper plants (*Capsicum annuum L*. cv. *Zunla-1.* from the Zunyi Academy of Agricultural Sciences) were first surface-sterilized with 10% hypochlorous acid, followed by three washes in sterile water. The plants were germinated in sterilized quartz sand until cotyledons fully emerged. The seedlings were grown for 15 d on full-strength nutrient solution with the following components: 1 mM NH_4_^+^, 4 mM NO_3_^−^, 2 mM K^+^, 1 mM Pi, 0.75 mM Ca^2+^, 0.5 mM Mg^2+^, 0.25 mM Cl^−^, 0.5 mM SO_4_^2−^, 20 µM Fe^2+^, 9 µM Mn^2+^, 46 µM BO_3_^3−^, 8µM Zn^2+^, 3 µM Cu^2+^, and 0.03 µM MoO_4_^2−^. For hydroponic experiments, the seedlings were grown in chamber programmed for 16 h of light at 28 °C and 8 h of dark at 20 °C, and supplied with nutrient solution including either 0.25 mM NH_4_^+^ or 2.5 mM NH_4_^+^ treatment.

For pot culture, three plantlets were transplanted to 3-dm^3^ pots filled with a 4:1 sand: soil mixture (the soil contained 90 mg kg^− 1^ available K, 1.5 mg kg^− 1^ NH_4_^+^, 3.1 mg kg^− 1^ NO_3_^−^, and 2.1 mg kg^− 1^ available Pi). A sand-based substrate containing *Rhizophagus irregularis* was used for AM inoculation. The irrigating solution contained 50 μm Pi to ensure a high AM colonization rate; 0.2 mM, 1 mM, 2 mM, or 5 mM NH_4_^+^; and other essential nutrients. The plants were harvested at 30 d post-inoculation for evaluation of the colonization rate, determination of the concentrations of N and P, and gene expression analysis.

A compartmentalized culture experiment was performed to evaluate mycorrhizal N uptake in chili pepper. The structure of the compartment culture system was designed as described by Liu et al. [16]. The seedlings were grown in root/fungal compartment and inoculated or non-inoculated with AM fungi. Two hyphal compartments contained a 4:1 sand: soil mixture with ^15^ N-labeled NH_4_^+^. The soil contained 90 mg kg^− 1^ available K, 1.5 mg kg^− 1^ NH_4_^+^, 3.1 mg kg^− 1^ NO_3_^−^, and 2.1 mg kg^− 1^ available Pi. The plants were irrigated with nutrient solution containing 2 mM NH_4_^+^, and 2 mM ^15^NH_4_^+^ (ammonium sulfate-^15^ N) were added to two hyphal compartments. After 5 weeks since inoculation, all the chili pepper plants were harvested and dried for 48 h at 70 °C; the methods for determining the ^15^ N content described by Wang [17] were used.

### **Identification of *CaAMT* genes in chili pepper plants**

The genome sequences of chili pepper were downloaded from NCBI (https://www.ncbi.nlm.nih.gov/genome/10896) [39]. The amino acid (AA) sequences of Arabidopsis and tomato AMTs were used as a query in BLASTP searches against the chili pepper genome with an E-value of 1.0E-10 to identify AMT genes. Hidden Markov model (HMM) profiles of the Pfam AMT domain (PF00909) were acquired from the Pfam database (http://pfam.sanger.ac.uk/family/PF00909) and used to search all chili pepper proteins with the Simple HMM Search tool in TBtools software [60]. The molecular weight (MW), number of AAs, and isoelectric point (pI) of each AMT protein were determined using the ExPasy online tool website (http://web.expasy.org/protparam/). WEBLOGO (http://weblogo.berkeley.edu/logo.cgi) was used to identify the conserved domains of the AMT subfamily.

### **Phylogenetic relationships, chromosomal distribution, and synteny analysis of *CaAMT* genes**

AMT protein sequences were aligned using ClustalX software with default gap penalties. An un-rooted phylogenetic tree was built using MEGA (version 7.0) software with the neighbor-joining (N-J) method based on the results of the multiple sequence alignment [61]. Bootstrap analysis was conducted with 1,000 replicates. The sequence data used in this study are provided in Supplemental Figure S1. The location of CaAMT genes on the chromosomes was determined from the genome annotation file using TBtools. The syntenic relationships of the AMT orthologs from chili pepper and other species were determined by constructing dual synteny plots using the MCScanX tool in TBtools software [60]. The nonsynonymous substitution ratios (*Ka*) and synonymous substitution ratios (*Ks*) of syntenic gene pairs were calculated using TBtools software.

### Histochemical β-glucuronidase (GUS) staining and detection of AM colonization

Histochemical staining of chili pepper roots to estimate GUS activity was performed following a previously described procedure [59]. The structure of mycorrhizae was characterized via co-localization of Magenta-GUS and trypan blue stains. Chili pepper roots stained with Magenta-GUS were treated with 10% KOH solution heated to 90 °C for 1 h; they were then counterstained with 0.3% trypan blue solution for 2 h at 90 °C. Excess stain from the stained materials was removed prior to photography by washing with 50% glycerol.

The magnified line-intersection method was used to quantify the degree of AM colonization. To determine the level of AM colonization, the percent root length colonization and the extent of AM colonization were determined at 10 randomly chosen spots on 10 stained 1-cm root segments using a microscope. At least three different root systems were examined in each treatment.

### Determination of the N and P concentration

Concentrations of N and P were measured from samples of chili pepper plants under different NH_4_^+^ levels. Briefly, dried samples were digested with 98% H_2_SO_4_ and 30% H_2_O_2_ following a previously described method [58]. The total N concentration in chili pepper plants was determined using the Kjeldahl method [62]. The total P concentration was determined via the molybdate-blue method following a method described by Chen et al. [58].

### Preparation of cDNA and quantitative real-time PCR (RT-PCR)

Total RNA of root and leaf samples was extracted using the Trizol reagent (Invitrogen); RNA samples were then treated with DNase I (Thermo Scientific™) to clear them of genomic DNA contamination. First-strand cDNA of each sample was generated using a reverse transcription kit (Thermo Scientific TM); the cDNA was then diluted threefold prior to RT-PCR reactions. All primers used for RT-PCR are listed in Table S3.

RT-PCR reactions were conducted in a total volume of 20 µL containing 10 µL of 2× One-Step SYBR Green Mix, 2 µL of cDNA, 0.4 µL of each primer, 0.4 µL of 50× ROX Reference Dye 1, and 6.8 µL of RNase-free ddH2O using an Applied Biosystems Plus RT-PCR System. Three technical replicates were performed for all RT-PCR reactions. The *Actin* gene was used to normalize the expression levels of each target gene[63]. The expression levels of AMT genes were calculated using the following formula: Y = 10^–(ΔCt/3)^ (ΔCt is the difference of the Ct between the *CaAMTs*/*SlAMTs* and the control *CaActin*/*SlActin* products) [64, 65].

### Binary vector construction and tomato transformation

To construct vectors with the promoters of CaAMT genes, a 1,112-bp promoter fragment of *CaAMT2;1* and a 1400-bp promoter fragment of *CaAMT2;2* immediately upstream of the ATG translation initiation codon were amplified using PCR. HindIII and KpnI restriction sites were then introduced at the end of the 5′ and 3′ regions. The construct with the promoter of the *CaAMT2;1* gene was referred to as *pCaAMT2;1*_*− 1112*_; the construct with the promoter of the *CaAMT2;2* gene was referred to as *pCaAMT2;2*_*− 1400*_. *Agrobacterium tumefaciens* strain EHA105-mediated transformation of tomato (*Solanum lycopersicum* cv. *Micro-Tom*, from the Weizmann Institute of Science, Rehovot, Israel) was conducted following a previously described procedure [66].

### Statistical analysis

The data means and standard errors (SE) were determined using Microsoft Excel 2019. Statistical significance between different plant genotypes and treatments was analyzed by One-Way ANOVA (IBM SPSS Statistics 25.0, Chicago, IL, USA) followed by a Turkey’s test (*P* < 0.05).

## Below is the link to the electronic supplementary material

**Additional file 1: Table S2.** Proteins sequence similarity matrix between members of CaAMT genes.

**Additional file 2: Table S3.** List of primers used in this study.

**Additional file 3: Table S1.** Detailed information about the CaAMT genes.

**Additional file 4: Figure S1.** Phylogenetic tree analysis of AMT family genes from pepper, eggplant, rice, tomato, *Arabidopsis*, potato, and Medicago. **Figure S2.** The predicted transmembrane domains of CaAMT proteins. **Figure S3.** Chromosomal location analysis of pepper AMT genes. The eight AMT genes were mapped to five different chromosomes using TBtools software. **Figure S4.** Effects of overexpression of CaAMT2.2 on tomato N accumulation under different NH4^+^ supply conditions.

## Data Availability

The genome sequence information of the eleven selected species were obtained from the National Center for Biotechnology Information websites. The data sets supporting the conclusions of this study are included within the article and its additional files.

## Declarations

### Statement

All methods were carried out in accordance with relevant guidelines and regulations.
