# Supplementary material for: Analysis of the AMT gene family in chili pepper and the effects of arbuscular mycorrhizal colonization on the expression patterns of CaAMT2 genes
Source: BMC Genomics. 2023 Mar 29;24:158. doi: 10.1186/s12864-023-09226-3 (PMC10053404; doi:10.1186/s12864-023-09226-3)
Supplement: Supplementary file 4 — Additional file 4: Figure S1. Phylogenetic tree analysis of AMT family genes from pepper, eggplant, rice, tomato, Arabidopsis, potato, and Medicago. Figure S2. The predicted transmembrane domains of CaAMT proteins. Figure S3. Chromosomal location analysis of pepper AMT genes. The eight AMT genes were mapped to five different chromosomes using TBtools software. Figure S4. Effects of overexpression of CaAMT2.2 on tomato N accumulation under different NH4+ supply conditions. [file 12864_2023_9226_MOESM4_ESM.docx]

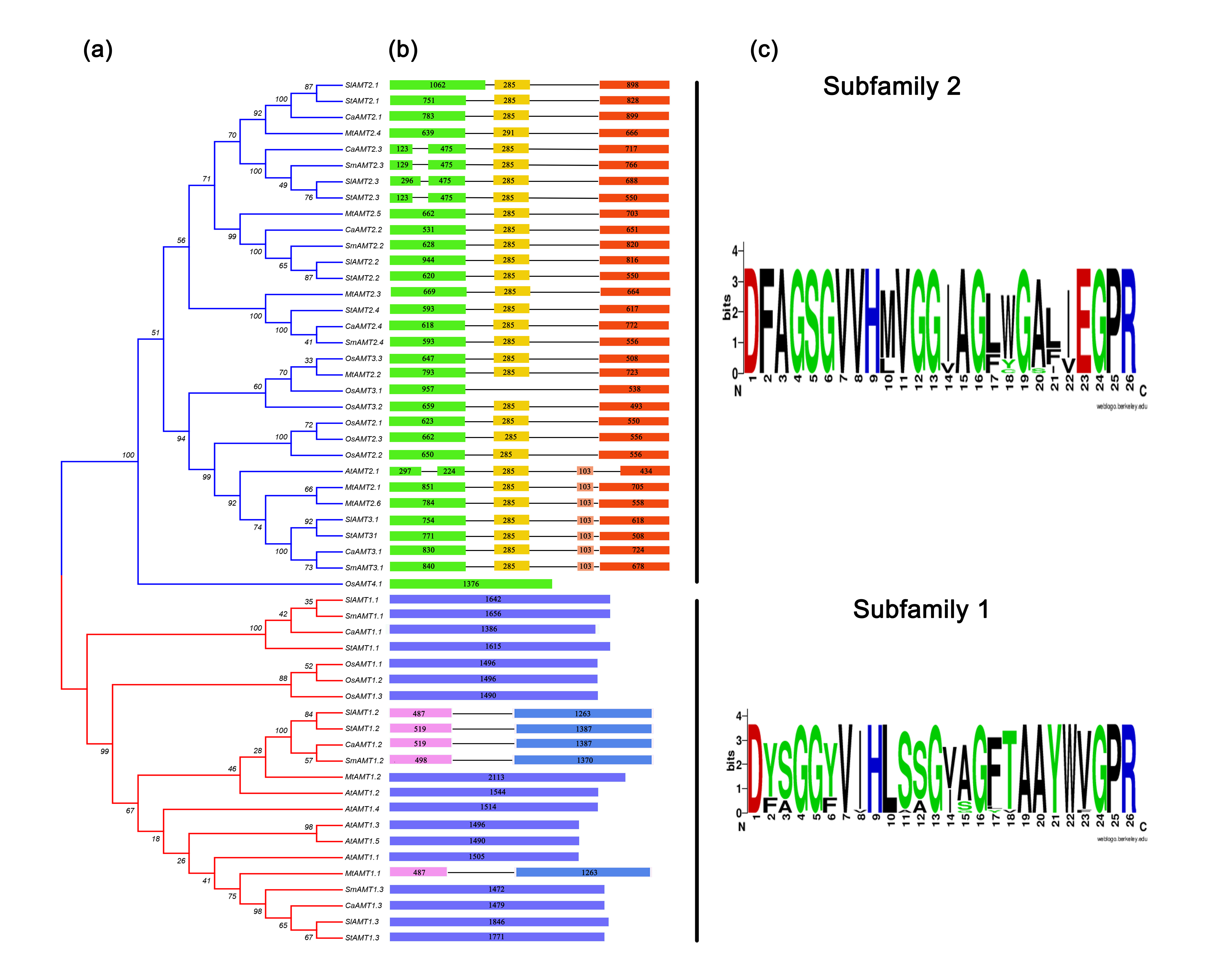


**Figure S1.** Phylogenetic tree analysis of AMT family genes from pepper, eggplant, rice, tomato, *Arabidopsis*, potato, and Medicago. (a) The phylogenetic tree of AMT proteins was constructed using the neighbor-joining method within the MEGA 7 software. (b) Exon-intron structures of the plant AMT genes. Exons and introns were displayed with colours boxes and black lines, respectively. (c) Two conserved domains of the AMT subfamilies were identified by TBtools software.


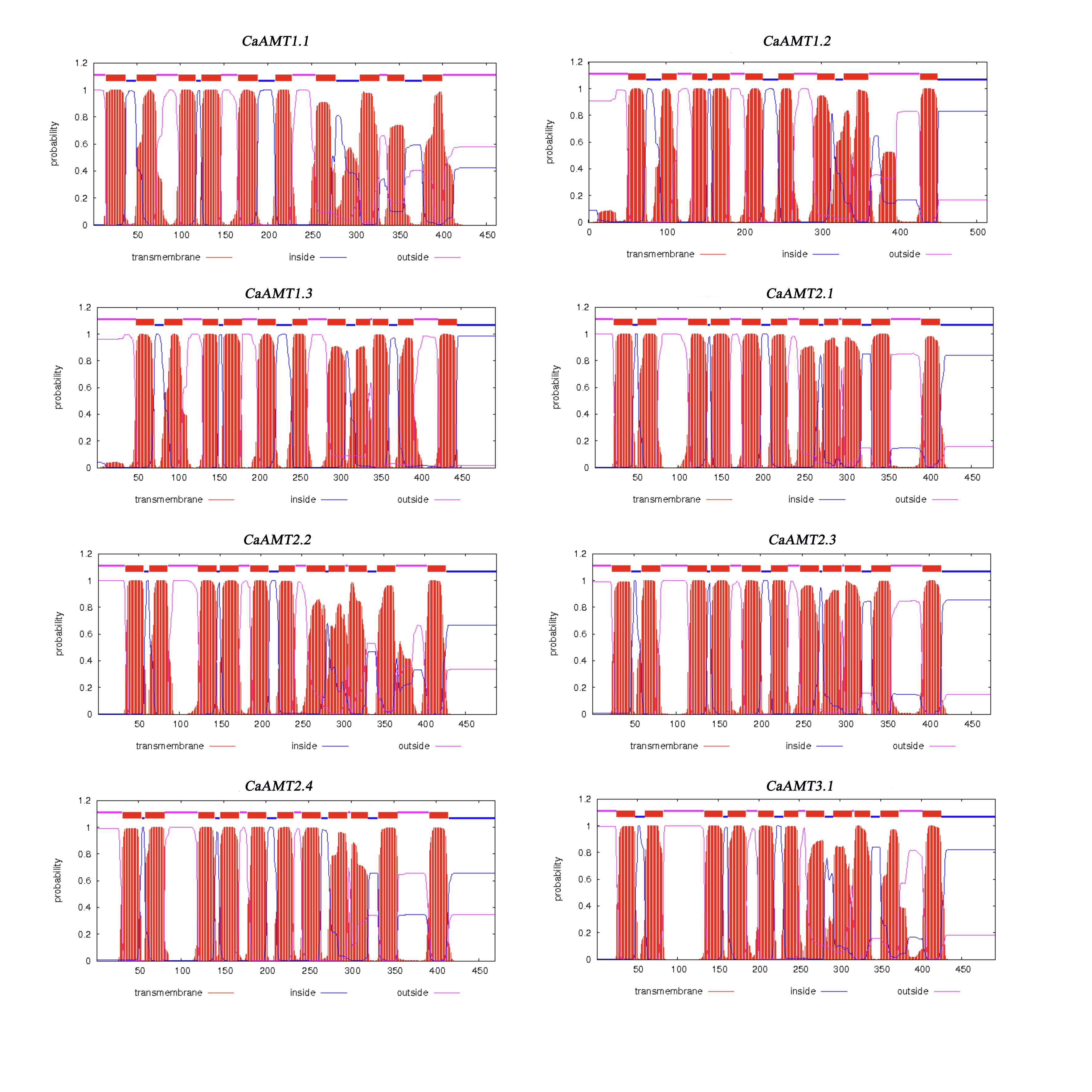


**Figure S2.** The predicted transmembrane domains of CaAMT proteins. TMHMM-2.0 (www.cbs.dtu.dk /services/TMHMM/) was used to predict the transmembrane domains, and red peaks represent the transmembrane regions.


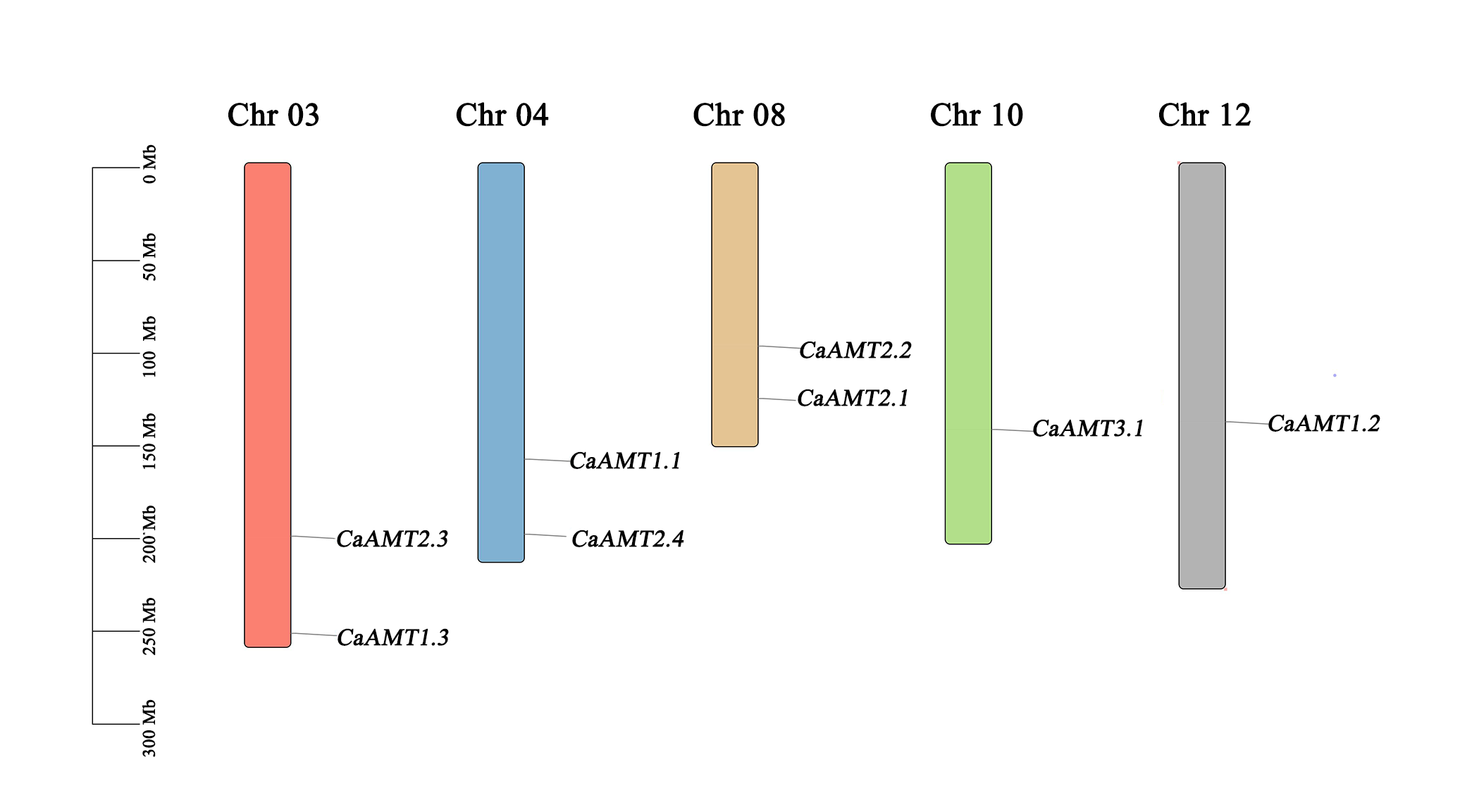


**Figure S3.** Chromosomal location analysis of pepper AMT genes. The eight AMT genes were mapped to five different chromosomes using TBtools software. The chromosomes ID are shown above each chromosome.


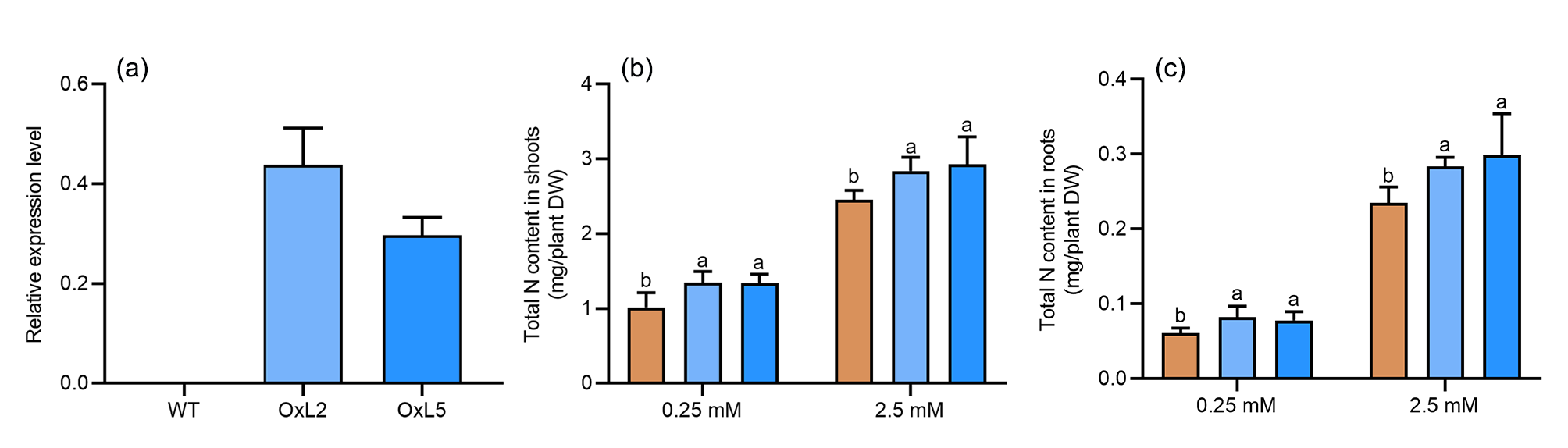


**Figure S4.** Effects of overexpression of CaAMT2.2 on tomato N accumulation under different NH_4_^+^ supply conditions. (a) Two transgenic lines (OxL2 and OxL5) which showed strong expression of CaAMT2.2 in tomato plants were selected. (b, c) The total N contents of in shoots and roots WT and transgenic lines were determined. Values are means ± SE of six biological replicates (n=6). Different letters indicate significant differences, *P* < 0.05.
